# Low‐Value Blood Culture Use in Adult Emergency Department Patients: A Scoping Review

**DOI:** 10.1111/1742-6723.70299

**Published:** 2026-06-28

**Authors:** Amy Weber, Upeksha Galappaththie, Joseph V. Moxon, Vinay Gangathimmaiah

**Affiliations:** ^1^ College of Medicine and Dentistry James Cook University Townsville Queensland Australia; ^2^ Department of Emergency Medicine Townsville University Hospital Townsville Queensland Australia

## Abstract

**Background:**

Blood cultures (BCs) are used in emergency departments (EDs) to investigate and manage suspected sepsis; however, their inappropriate use contributes to low‐value care, providing limited clinical benefit while causing avoidable harm. Despite recommendations for more selective testing, low‐value blood culture (LVBC) use persists, and there remains uncertainty on the definition, drivers, and impact of LVBCs.

**Objectives:**

To map the existing literature on LVBCs in adult ED patients, including definitions of LVBCs, associated clinical contexts, contributing factors, consequences and related gaps in knowledge.

**Methods:**

Six databases were searched from inception to June 30, 2025. Studies involving adult populations and addressing LVBC use in EDs were included. Data were charted independently by two reviewers. Risk of bias was assessed using JBI critical appraisal tools.

**Results:**

Of 1453 records screened, 18 studies were included in this scoping review. None defined LVBCs, although five themes characterising LVBCs emerged: limited impact on patient management, non‐adherence to guidelines, failure to meet predictive tool thresholds, protocol‐driven ordering, and BCs deemed unnecessary by clinicians. Reported prevalence of LVBCs ranged from 19% to 69%. The most referenced LVBC driver was pressure to comply with quality measures. Reported consequences of LVBCs included unnecessary treatment, investigation and prolonged stay due to false positive results. Patient‐centred outcomes were rarely reported, and formal economic evaluations were lacking.

**Conclusion:**

LVBCs are prevalent among adult ED patients yet inconsistently defined. Addressing the issue will require standardised criteria, interventions targeting root causes, and research focused on patient‐centred and economic consequences to optimise BC ordering practices.

## 1 Introduction

### 1.1 Rationale

Low‐value care describes healthcare which provides minimal benefits to patients, poses a risk of patient harm, or involves costs disproportionate to benefits [1, 2]. Blood cultures (BCs) are frequently used in emergency departments (EDs) to identify bloodstream infections such as bacteraemia [3], and guide targeted antimicrobial therapy in patients with suspected sepsis. While invaluable in these contexts, overuse may constitute low‐value care, providing minimal clinical benefits, exposing patients to avoidable harm, and straining resources [4, 5, 6].

The Australasian College for Emergency Medicine recommends against BCs for patients who are not systemically septic, have a clear infection source, and where direct sampling for culture (e.g., urine) is possible [7]. However, low‐value blood culture (LVBC) use persists [4], driven by a variety of systemic, behavioural and clinical factors [8, 9, 10, 11]. Inappropriate BCs lead to low true‐positive rates and minimal impact on patient management. False positive results due to contamination cause additional harm, including unnecessary antibiotics, investigations and prolonged hospital stays, compounded by the avoidable healthcare costs and strain on the healthcare system [12, 13, 14, 15].

To date, no comprehensive synthesis of the literature surrounding LVBCs within adult ED populations has been conducted. Additionally, existing literature does not explicitly define LVBCs, although it uses proxy measures allowing post hoc interpretation and evaluation. There is currently no consistent understanding of the nature, causes, and impacts of LVBCs. A scoping review is warranted to systematically map how LVBCs are characterised in the literature, the settings in which they occur, and the consequences of their use. This will clarify concepts and identify gaps for future research and quality improvement.

### 1.2 Objectives

This review aims to map existing literature on LVBCs in adult EDs, framed by the research question: “What is known from existing literature about LVBC use in adult ED patients?”

The specific objectives are to:

1. Describe criteria used to define LVBCs in the emergency department.
2. Examine clinical contexts and scenarios in which BCs are of low value.
3. Summarise reported outcomes associated with LVBC use, including the frequency/prevalence, harms and costs.
4. Identify gaps in the literature and highlight areas for possible future research.

## 2 Methods

This scoping review was conducted according to the Joanna Briggs Institute (JBI) methodological framework for scoping reviews [6]. Reporting followed the Preferred Reporting Items for Systematic reviews and Meta‐Analyses extension for Scoping Reviews (PRISMA‐ScR) guideline [16] (Appendix 1, Supporting Information).

### 2.1 Protocol and Registration

A protocol was registered with Open Science Framework (OSF) Registries, accessible at https://doi.org/10.17605/OSF.IO/6XTZ3.

### 2.2 Eligibility Criteria

Study selection was guided by the Population, Concept, Context (PCC) framework [17]. Studies were included if they examined BCs in adult patients (≥ 18 years) within ED settings and addressed concepts consistent with low‐value care. Primary quantitative, qualitative, and mixed‐methods studies were eligible. Grey literature was excluded to focus on peer‐reviewed, empirical evidence. Eligibility criteria are detailed in Appendix 2, Supporting Information.

### 2.3 Information Sources and Search Strategy

MEDLINE, Embase, Emcare (Ovid), CINAHL, Scopus and Web of Science were searched from inception to 30 June 2025. Reference lists of included studies and identified reviews were hand‐searched. The search strategy was developed in collaboration with a health sciences librarian. Search terms incorporated synonyms for “blood cultures”, “low‐value care”, and “emergency department”. Complete search strategies are detailed in Appendix 3, Supporting Information.

### 2.4 Selection of Sources of Evidence

Search results were imported into EndNote 21 [18] for removal of duplicates, then exported to JBI System for Unified Management, Assessment and Review of Information (SUMARI) [19] for screening. Reviewers (AW and UG) independently performed title and abstract screening, and full text screening. Discrepancies were resolved through discussion or a third reviewer (VG or JM). Several studies [20, 21, 22, 23, 24, 25, 26, 27, 28] did not specify ages of their participants. In such cases, corresponding authors were contacted to confirm participants were adults ≥ 18 years. Studies were excluded where population eligibility could not be confirmed. The selection process was recorded using a PRISMA diagram [29].

### 2.5 Operational Definition of LVBCs

LVBCs were not explicitly defined in any studies involved in full text screening. For the purposes of this review, LVBCs were operationally defined as BCs aligning with low‐value care principles. Classification of BCs as ‘low value’ occurred post hoc by review authors, representing an interpretive synthesis based on reported study outcomes. This classification is not to be interpreted as an outcome directly reported in primary studies.

### 2.6 Data Charting Process

Data items were independently charted by two reviewers (AW and UG) using an Excel spreadsheet (Microsoft 365) [30]. Data extracted included basic study characteristics, interpreted criteria used to define LVBCs, their prevalence, associated clinical features, contributing factors and consequences of LVBCs. Additional data included BC outcomes, study limitations and author recommendations. (Appendix 4, Supporting Information).

### 2.7 Critical Appraisal of Individual Sources of Evidence

Studies were critically appraised using JBI Critical Appraisal Tools [31, 32, 33, 34] appropriate to study designs by two independent reviewers (AW and UG), with discrepancies resolved by consensus. The proportion of applicable criteria met was calculated for each study. While JBI does not provide thresholds for categorising risk of bias, for the purpose of this review, studies were classified as having low risk of bias if ≥ 80% of applicable checklist criteria were met, moderate risk if 60%–79% were met, and high risk if < 60% were met [35]. Studies were included regardless of their risk of bias.

### 2.8 Synthesis of Results

A narrative synthesis was conducted aligned with JBI scoping review methodology, using qualitative analysis. Results were organised into themes, including definitions/criteria and prevalence of LVBCs, associated clinical contexts, drivers of use and associated harms, including economic implications. Findings were presented narratively using tables and figures. Critical appraisal findings were considered to contextualise results.

## 3 Results

### 3.1 Selection of Sources of Evidence

**FIGURE 1:**
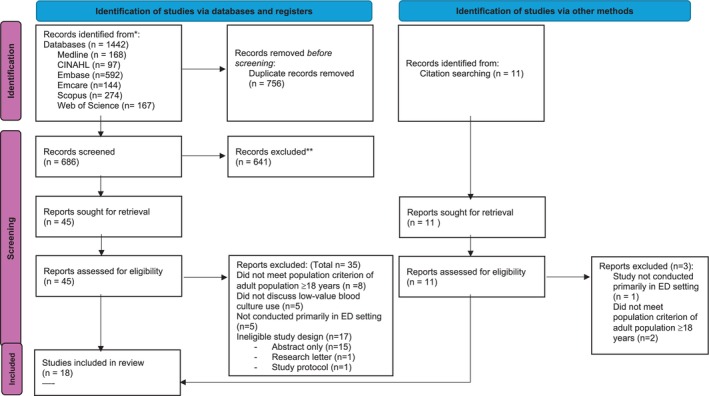
PRISMA 2020 flow diagram for new systematic reviews which included search of databases, registers and other sources.

A total of 1442 records were identified via database search. After removing duplicates, 686 unique records were screened by title and abstract, of which 45 underwent full‐text screening. Ten studies met inclusion criteria, with an additional eight studies identified through citation searching of key articles. 18 studies were included in the review (Figure 1) [29].

### 3.2 Characteristics of Sources of Evidence

The 18 studies included were published between 1998 and 2024 (Table 1). Majority were conducted in the United States (*n* = 9) [23, 36, 38, 40, 42, 43, 44, 47, 49], followed by the Netherlands (*n* = 4) [20, 39, 46, 48] (including two multinational studies involving Spain [39] and the United States [46]), Australia (*n* = 2) [22, 37], Germany (*n* = 2) [21, 45] and Switzerland (*n* = 1) [41]. Study durations ranged from 2 months [49] to 10 years [46], with sample sizes from 183 patients [45] to over 25,000 BC events [47].

**TABLE 1:** Characteristics of studies included in scoping review specifically exploring low‐value blood cultures in adult emergency department patients.

Study author(s) (Year)	Country	Study design	Population characteristics^a^	Sample size	Study focus
Benenson et al. (2007) [36]	United States	Retrospective chart review	Hospitalised with pneumonia	909	Utility of BCs in pneumonia patients
Boerman et al. (2022) [20]	Netherlands	Retrospective observational	Suspected bacterial infection	1487^b^	Developing predictive model for bacteraemia/BC outcomes
Brown et al. (2016) [37]	Australia	Retrospective chart review	Immunocompetent	3931^c^	Utility of modified Shapiro rule in reducing negative BCs Clinical impact of positive BCs
Corbo et al. (2004) [38]	United States	Retrospective observational cohort	Immunocompetent, hospitalised with CAP, BCs before antibiotics	355	Proportion of false positive versus true positive BCs Frequency of change in antibiotic therapy based on BC results.
Ehrenstein et al. (2005) [21]	Germany	Prospective observational	Hospitalised > 3 days	188	Consequences of BC results in a population commonly treated empirically with broad spectrum antibiotics
Kaal et al. (2024) [39]	Netherlands and Spain	Prospective observational cohort	No additional	Derivation: 2111 Validation: 4436	Development and external validation of a bacteraemia prediction model incorporating PCT
Kelly (1998) [22]	United States	Retrospective chart review	No additional	1062	Proportion of positive BCs BCs impacting patient management Guideline development for the appropriate ordering of BCs
Kennedy et al. (2005) [40]	United States	Prospective observational cohort	Pneumonia with BCs collected	414	Impact of ED BCs on antimicrobial therapy for patients with pneumonia
Laukemann et al. (2015) [41]	Switzerland	Prospective observational cohort	Suspected infection	1083	Comparison of five different clinical scores and six biomarkers for their ability to predict BC positivity
Makam et al. (2014) [42]	United States	Retrospective repeated cross‐sectional	Hospitalised with CAP (ICD codes)	1487^b^	Patterns of obtaining BCs in adults hospitalised with CAP
Makam et al. (2015) [43]	United States	Retrospective repeated cross‐sectional	Non‐pneumonia respiratory illness (ICD codes)	4854^b^	Frequency of BCs ordered among patients hospitalised with non‐pneumonia related respiratory symptoms
Paolo et al. (2013) [44]	United States	Retrospective chart review	Cases‐ complicated cellulitis Controls‐ standard cellulitis^d^	639	Role of BCs in management of complicated cellulitis
Pawlowicz et al. (2016) [23]	Australia	Retrospective observational pre and post intervention	No additional	16887^c^	Impact of implementation of BC ordering algorithm on ordering and contamination rates Indications providers cite for BCs collected in ED
Rothe et al. (2020) [45]	Germany	Retrospective observational cohort	Suspected urinary tract infection with blood culture, urine culture and procalcitonin tested	183	Diagnostic criteria for systemic UTI and collection of PCT and paired urine blood samples
Schinkel et al. (2022) [46]	Netherlands and United States	Retrospective observation with prospective evaluation	No additional	44,123^c^, ^e^	Simplifying and validating developed machine learning model to reduce BCs in low‐risk patients.
Theophanous et al. (2024) [47]	United States	Quasi‐experimental pre and post intervention	No additional	25242^c^	Improve diagnostic stewardship through appropriate emergency department blood culture ordering and use
Van Nieuwkoop et al. (2010) [48]	Netherlands	Prospective observational cohort	Febrile urinary tract infection with blood cultures and procalcitonin	581	Clinical characteristics PCT value to predict bacteraemia in febrile UTI
Zitek et al. (2020) [49]	United States	Retrospective chart review	Code sepsis initiated in ED	544	Proportion of patients undergoing a 1‐h sepsis protocol diagnosed with sepsis and rate of positive BCs

Abbreviations: BC, blood culture; CAP, community acquired pneumonia; ED, emergency department; ICD, International Classification of Diseases; PCT, procalcitonin; UTI, urinary tract infection.

^a^ Characteristics in addition to: adult patients with blood cultures obtained in emergency department.

^b^ Emergency department visits (number of patients included not reported).

^c^ Number of blood cultures studied (number of patients included not reported).

^d^ Complicated cellulitis defined as patients with cellulitis and any of: active chemotherapy, dialysis, human immunodeficiency virus/acquired immune deficiency syndrome, diabetes, or organ transplantation. Standard cellulitis defined as patients with cellulitis without a priori defined comorbidities—who presented to the ED during the same time period.

^e^ Total blood cultures including sampling in the Amsterdam UMC (locations VUMC and AMC; the Netherlands), Zaans Medical Center (ZMC; the Netherlands), and Beth Israel Deaconess Medical Center (BIDMC; United States).

Study designs varied, including nine retrospective studies (chart reviews [22, 36, 37, 44, 49], observational cohorts [20, 38, 45], observational pre‐post intervention study [23]), five prospective observational cohort studies [21, 39, 40, 41, 48], two repeated cross‐sectional analyses [42, 43], one quasi‐experimental pre‐post intervention study [23] and one study combining retrospective observation with prospective evaluation [46]. Some studies focused on specific presentations including pneumonia [36, 38, 40, 42], suspected sepsis [20, 41], and urinary tract infections (UTIs) [45, 48]. Aims varied, although generally focused on investigating BC ordering practices, outcomes, clinical utility, and evaluating/validating predictive tools to support BC ordering. None explicitly focused on LVBCs, although all addressed concepts aligned with low‐value care [20, 36, 38, 40, 41, 42, 45, 48].

### 3.3 Critical Appraisal Within Sources of Evidence

Fifteen studies [20, 22, 23, 36, 37, 38, 39, 40, 41, 44, 45, 46, 47, 48, 49] met ≥ 80% of the applicable checklist criteria, indicating low risk of bias. The remaining three studies [21, 42, 43] met 70%–75% of criteria, hence of moderate risk of bias (defined as 60%–79%). No studies were assessed as high risk (< 60%). A common limitation was the potential misclassification of BC results as true versus false positives [20, 38, 42, 46]. Several studies reported additional limitations affecting generalisability including retrospective design, single‐centre settings, and limited subgroup analyses, detailed in the tool comments. These suggest caution when extrapolating results to broader populations and settings (Table 2).

**TABLE 2:** Summary of critical appraisal results for included studies using Joana Briggs Institute Critical Appraisal Checklists.

Study Author(s) (Year)	JBI critical appraisal checklist	Criteria met/Total applicable	% score	Risk of bias	Comments
Benenson et al. (2007) [36]	Case Series [31]^a^	10/10	100	Low	Limited generalisability to all groups at risk of multidrug resistant pathogens. Possible inter‐rater variability in data collection Small number of patients in some subgroups/with true positives
Boerman et al. (2022) [20]	Diagnostic Test Accuracy [32]	8/10	80	Low	Possible misclassification of positive versus contaminated BCs Lack of potentially predictive variables and free text data extracted from electronic records Single centre, requires further external validation
Brown et al., (2016) [37]	Diagnostic Test Accuracy [32]	9/10	90	Low	No blinding of data collector to the aims Retrospective bias Single centre study, limited generalisability
Corbo et al. (2004) [38]	Case Series [31]^a^	8/10	80	Low	Subjective classification of ‘contaminants’ by physicians Retrospective design, no insight into reasons why antibiotics were changed
Ehrenstein et al. (2005) [21]	Case Series [31]^a^	7/10	70	Moderate	ED physician discretion used in BC decision making without infectious disease consult
Kaal et al. (2024) [39]	Diagnostic Test Accuracy [32]	10/10	100	Low	Unexplained geographic variation in model performance
Kelly, 1998 [22]	Case Series [31]^a^	9/10	90	Low	No comments
Kennedy et al. (2005) [40]	Case Series [31]^a^	9/10	90	Low	Did not account for patient comorbidities‐possible overestimation of utility of BCs in all ED pneumonia patients
Laukemann et al. (2015) [41]	Diagnostic Test Accuracy [32]	10/10	100	Low	Higher risk study population likely to have higher true positive BC rate.
Makam et al. (2014) [42]	Cross‐sectional Analysis [34]	6/8	75	Moderate	Omission of data from 2005 through 2006 limiting trend analysis Possible misclassification of BC use
Makam et al. (2015) [43]	Cross‐sectional Analysis [34]	6/8	75	Moderate	Omission of 2005–2006 data limits trend analysis Potential misclassification/under coding in NHAMCS Unable to evaluate culture appropriateness or contamination rates, no clinical detail on culture yield or outcomes
Paolo et al. (2013) [44]	Cohort [34]	8/10	80	Low	Retrospective design, results predicated by provided documentation Majority of cohort diabetic patients, smaller representation of immunocompromised group Immunocompromised defined by documentation of treating provider (no further evidence)
Pawlowicz et al. (2016) [23]	Quasi‐experimental [33]	8/9	89	Low	Retrospective single centre design Aggregate data only, unable to exclude duplicate culture orders Inconsistent documentation of indications for obtaining BCs
Rothe et al. (2020) [45]	Diagnostic Test accuracy [32]	8/10	80	Low	Single centre design limiting generalisability Retrospective design, no additional details on quality of BCs/clinical end points available Strict inclusion criteria limiting representation of ED presentations
Schinkel et al. (2022) [46]	Diagnostic Test Accuracy [32]	9/10	90	Low	Performance of tool in subgroups with specific comorbidities or medications not examined Possible misclassifications of microorganisms as pathogens versus contaminants in specific groups Model performance may vary over time due to changes in patient characteristics/prevalence of positive cultures
Theophanous (2024) [47]	Quasi‐experimental [33]	8/9	89	Low	Single centre setting without control Reviewers unblinded at start of intervention Started adjudication for true positive versus contaminant later in analysis, possible skewing of results Resource intensive intervention with limited generalisability.
Van Nieuwkoop (2010) [48]	Diagnostic Test Accuracy [32]	8/10	80	Low	Frequent pre‐treatment antibiotic use at presentation, potential for false negatives Measurement of procalcitonin values after BC performed and measurement in routine clinical practice may differ (i.e., use of point of care assay)
Zitek (2020) [49]	Case Series [31]^a^	8/10	80	Low	Retrospective design and possible unmeasured confounders Possible incomplete documentation of diagnoses resulting in falsely low results Limited generalisability for other populations/settings No data available for patients undergoing 3‐h sepsis bundle, limiting comparability

Abbreviations: BC, blood cultures; ED, emergency department; JBI, Joanna Briggs Institute.

^a^ Case series checklist‐ although formal case series studies were excluded from this scoping review, the Joanna Briggs Institute (JBI) Case Series Checklist was deemed the most appropriate tool for retrospective chart reviews. These studies were typically single‐arm observational designs without a control group and lacked comparison data, aligning more closely with the case series checklist structure than other JBI tools.

### 3.4 Criteria for LVBCs and Reported Prevalence

No studies explicitly defined LVBCs. Instead, BCs were described using terms such as “limited utility,” [36, 42] “unnecessary,” [23, 39, 46] “inappropriate” [47], “limited usefulness,” [38] or “avoidable.” [48] While the term “low‐value” was absent, such descriptions of BCs in these studies aligned with low‐value care. Criteria used to characterise BCs interpreted as low‐value were hence identified and categorised into five themes (Table 3):

1. Limited impact on patient management (i.e., antibiotic therapy) [21, 36, 38, 40, 44]‐ particularly in community acquired pneumonia (CAP).
2. Did not meet criteria for BC collection using prediction models, algorithms, scoring tools [20, 23, 37, 39, 41, 45, 46, 47, 48]‐ BCs ordered despite low predicted risk of bacteraemia.
3. Non‐adherence to guideline recommendations [22, 42].
4. Deemed unnecessary according to clinical judgement [21]‐ as per retrospective assessment of the impact on patient care by the treating clinician.
5. Non‐selective protocol‐driven overuse [49]‐ BCs ordered based on standardised protocols regardless of individual clinical risk.

**TABLE 3:** Criteria, prevalence estimates, and clinical outcomes associated with low‐value blood cultures in adult emergency department patients.

Theme of criteria used to determine LVBCs	Study Author (Year)	Criteria for low‐value blood cultures	Estimated prevalence of LVBC use, %	% BCs impacting patient management, %	Additional relevant findings
Limited impact on patient management	Benenson et al. (2007) [36]	Ordered in CAP patients, resulting in limited changes in antibiotic therapy.	NR	0.04	
	Corbo et al. (2004) [38]	Ordered in immunocompetent CAP patients resulting in limited changes in antibiotic therapy.	NR	5	False positives approximate true positives Therapy changes not driven by detection of resistant organisms.
	Ehrenstein et al. (2005) [21]	Multiple criteria suggested (1) No impact on patient management (2) Retrospectively deemed unnecessary by treating physicians, after viewing results, to (a) elucidate infection aetiology and (b) decide antibiotic therapy	(1) N/A (2) a‐54 (3) b‐55	11	Positive cultures triggered antibiotic adjustments in ~50% of cases. Negative results rarely changed management.
	Kennedy et al. (2005) [40]	Ordered in pneumonia patients, rarely altering antibiotic therapy.	NR	3.6	Change in treatment based on resistance patterns infrequent Therapy often not narrowed despite the availability of evidence to support
	Paolo et al. (2013) [44]	Ordered in patients with complicated cellulitis, rarely impacting patient management.	NR	2	
Did not meet criteria for blood culture collection using prediction models, algorithms, scorings tools	Machine Learning Model				
	Boerman et al. (2022) [20]	Did not meet criteria of machine learning prediction models (gradient boosted trees and logistic regression).	69^a^	NR	Two predictive models achieved AUROCs of 0.77 and 0.78. Well suited for implementation in practice.
	Schinkel et al. (2022) [46]	BCs which would be avoided due to low (< 5%) risk of a positive results, according to XGBoost machine learning model.	30.3	NR	
	Clinical Score or Algorithm				
	Brown et al. (2016) [37]	Did not meet Modified Shapiro Rule threshold (score of 2 or more) in immunocompetent patients.	48	3.4	Modified Shapiro Rule safe and effective in predicting bacteraemia in ED patients.
	Laukemann et al. (2015) [41]	Did not meet criteria for BC collection using Shapiro criteria (≥ 3) or PCT > 0.25 μg/L.	40	NR	
	Pawlowicz et al.(2016) [23]	Did not meet criteria of BC collection algorithm (involving immunocompromised status, Shapiro rule, CAP culture criteria^c^)	33.3	NR	Institutional protocols can be implemented quickly and be efficacious in preventing unnecessary BCs
	Rothe et al. (2020) [45]	Did not meet the criteria of algorithm: ‘3Fs score’^b^ and/or procalcitonin < 0.25 pg/mL.	33.3	NR	Algorithm could lead to a reduction of unnecessary BCs and provide a timely, cost‐effective and safe option for ED patients.
	Theophanous et al.(2024) [47]	Not required as per decision‐support algorithm identifying features of clinical instability/high probability of bacteraemia.	19	NR	Reduction in BC events after algorithm implementation, improved BC outcomes and sustained response post cessation of chart review. No increase in adverse safety events
	Biomarker‐Based Prediction Tool				
	Kaal et al. (2024) [39]	Deemed inappropriate according to basic model with procalcitonin, using < 5% risk threshold for bacteraemia.	29	NR	Model implementation could have saved 29% of BCs while only missing 1.1% of true positive BCs.
	Laukemann et al. (2015) [41]	See above in “Clinical Risk Score”			
	Van Nieuwkoop et al. (2010) [48]	Deemed avoidable by prediction model in febrile urinary tract patients with a preset procalcitonin cutoff value of less than or equal to 0.25microg/L.	40	NR	Level of PCT appeared to be a marker of the bacterial load in bacteraemia cases
	Rothe et al. (2020) [45]	See above in “Clinical decision support score/algorithm”			
Non‐adherence to guideline recommendations	Kelly (1998) [22]	Did not meet guidelines suggested by author: (1) likely bacterial infection, (2) no possible alternative direct specimen for culture (e.g., sputum), (3) inpatient treatment warranted	40	1.7	
	Makam et al. (2014) [42]	Ordered in all patients hospitalised with CAP, despite guideline recommendations (IDSA/ATS 2007 guidelines)^d^	NR	NR	Non‐clinical factors were powerful predictors of BC use (i.e., hospital ownership and region).
	Makam et al. (2015) [43]	Ordered in hospitalised patients with respiratory symptoms not attributable to pneumonia despite guideline recommendations (IDSA/ATS 2007 guidelines)^d^	NR	NR	BC collection in patients with non‐pneumonia respiratory symptoms increased in parallel with to use in CAP
Deemed unnecessary by physician according to clinical judgement	Ehrenstein et al. (2005) [21]	See above in “Limited impact on patient management”			
Non‐selective protocol driven overuse	Zitek et al. (2020) [49]	Ordered in all patients with code sepsis initiated as part of the 1‐h sepsis bundle	NR	1.5	

Abbreviations: BC, blood culture; CAP, community acquired pneumonia; N/A, not applicable; NR, Not reported.

^a^ Percentage of blood cultures avoided using gradient boosted trees model.

^b^ 3F score for diagnosis of urinary tract infections: ‘Fever’, ‘failure’ (organ dysfunction) and ‘focus’ (focal urinary tract symptoms).

^c^ ‘CAP Culture Criteria’ met if patient has any one of: anticipated admission to intensive care unit or IMC within 24 h, leukopenia, chronic severe liver disease, pleural effusion or active alcohol abuse.

^d^ Infectious Disease Society of America/American Thoracic Society Consensus Guidelines on management of community acquired pneumonia in adults recommending blood culture collection for patients with severe community acquired pneumonia.

Only studies employing predictive tools or guideline‐based criteria reported estimates of unnecessary BCs, typically by identifying the proportion of BCs that their intervention could safely avoid or reduce. These estimates were interpreted by the authors as proxy measures of LVBC prevalence and varied widely from 19% [47] to 69% [37], depending on the population studied.

### 3.5 Impact on Patient Management

Eight studies reported the impact of BCs on patient management [21, 22, 36, 37, 38, 40, 44, 48]. From a cumulative total of 7854 BCs, 223 (2.8%) were reported to directly influence patient management, prompting changes in antibiotics. Two studies [36, 40] noted that even when BC results suggested adjustments in antibiotics based on sensitivities, changes were not always implemented.

### 3.6 BC Yield

The proportion of true‐positive BC results ranged from 1.2% [47] to 20% [39]. In many cases, false positives due to contamination equalled or exceeded true positives [36, 37, 38]. Table 4 summarises the outcomes and impact of BCs studied (Table 4).

**TABLE 4:** Diagnostic yield, contamination rates, and clinical impact of blood cultures in studies examining low‐value use in adult emergency department patients.

Study Author(s) (Year)	Total blood cultures, *n*	Total positive blood cultures, *n* (%)	True positive blood culture results, *n* (%)	False positive/contaminated blood culture results, *n* (%)	Blood culture results impacting patient management, *n* (%)
Benenson et al. (2007) [36]	684	77 (11.3)	23 (3.4)	54 (7.9)	3 (0.04)
Boerman et al. (2022) [20]	4885	NR	598 (12.2)	254 (5.2)	NR
Brown et al. (2016) [37]	3931	373 (9.5)	151 (3.8)	222 (5.6)	133 (3.4)
Corbo et al. (2004) [38]	355	70 (20)	33 (9)	37 (10)	18 (5)
Ehrenstein et al. (2005) [21]	188	48 (25.5)	24 (12.8)	23 (12.2)	15 (8)^e^
Kaal et al. (2024) [39]	Derivation: 2980	NR	273 (13)	84 (2.8)	NR
	External validation: 4436	NR	896 (20)	227 (5.1)	NR
Kelly (1998) [22]	1062	92 (8.7)	52 (4.9)	40 (3.8)	18 (1.7)
Kennedy et al. (2005) [40]	414	NR	29 (7)	25 (17.36)	15 (3.6)
Laukemann et al. (2015) [41]	1083	NR	104 (9.6)	28 (2.6)	NR
Makam et al. (2014) [42]	~623^a^	NR	NR	NR	NR
Makam et al. (2015) [43]	~672^b^	NR	NR	NR	NR
Paolo et al. (2013) [44]^c^	639	46 (7.2)	25 (4)^e^	23 (3.6)	10 (1.6)
Cases	314	29 (9.2)	NR	13 (4.1)	6 (1.9)
Controls	325	17 (5.2)	NR	10 (3.1)	4 (1.2)
Pawlowicz et al. (2016) [23]	16,887	NR	NR	NR	NR
Rothe et al. (2020) [45]	183	NR	NR	NR	NR
Schinkel et al. (2022) [46]^d^	VUMC Training: 6421	NR	73,841 (11.5)^e^	404 (6.3)^e^	NR
	VUMC Test: 1606		18,469 (11.5)^e^	101 (6.3)^e^	
	AMC Validation: 2429		27,204 (11.2)^e^	257 (10.6)^e^	
	ZMC Validation: 5961		733 (12.3)^e^	309 (5.2)^e^	
	BIDMC Validation: 27706		1496 (5.4)^e^	1357 (4.9)^e^	
Theophanous et al. (2024) [47]	1730	NR	194 (1.2)	74 (4.2)	NR
Van Nieuwkoop et al. (2010) [48]	581	NR	NR	16 (3)	NR
Zitek et al. (2020) [49]	541	75 (13.9)	38 (7)	37 (6.8)	24 (1.5)

Abbreviation: NR, not reported.

^a^ Total number of blood cultures collected not explicitly stated, although reported that blood cultures were collected in 41.9% of 1487 total visits to ED by patients hospitalised for community acquired pneumonia from 2002 to 2010, therefore ~623 blood cultures collected.

^b^ Total number of blood cultures collected not explicitly stated, although reported that blood cultures were collected in 9.8% of 2175 total ED visits studied during 2002–2004, 14.1% of 1346 visits during 2007–2008, and 19.9% of 1333 visits during 2009–2010. Hence ~672 blood cultures collected in total during study period.

^c^ Cases: complicated cellulitis cohort including individuals with diabetes mellitus, who are immunocompromised (human immunodeficiency virus/acquired immune deficiency syndrome, in active chemotherapy status post solid organ transplantation), or with peripheral vascular insufficiency. Controls: patients with standard cellulitis—without a priori defined comorbidities—who presented to the ED during the same period.

^d^ Multicentre study, AMC = Academic Medical Center; BIDMC = Beth Israel Deaconess Medical Center; VUMC = VU Medical Center; ZMC = Zaans Medical Center.

^e^ Number of blood cultures not explicitly stated, *n* calculated from % and total number provided.

True positives were generally defined as the growth of pathogens consistent with the suspected infection [22, 40, 41, 44, 49]. Cultures were considered false positive if they grew organisms deemed contaminants [21, 36, 40, 44, 46, 48]. Determinations of contamination were informed by: microbiology guidelines [36, 40], prior literature [44, 46], or the clinical judgement of treating physicians/microbiologist [22, 38]. Some studies used predefined lists to define true pathogens and contaminants [21, 46, 49].

### 3.7 Correlation Between Yield and Clinical/Laboratory Criteria

Clinical and laboratory features commonly used to guide BC decisions showed limited predictive value for positive BCs. Height of fever [22], leucocytosis [22, 47], neutrophil count [22], and isolated inflammatory biomarkers such as C‐reactive protein, red blood cell distribution width, and white blood cell count [41] did not correlate with positivity. Machine learning models associated higher potassium and lymphocytes with lower risk of bacteraemia, although no feature alone reliably predicted yield [46].

### 3.8 Drivers of LVBCs

**FIGURE 2:**
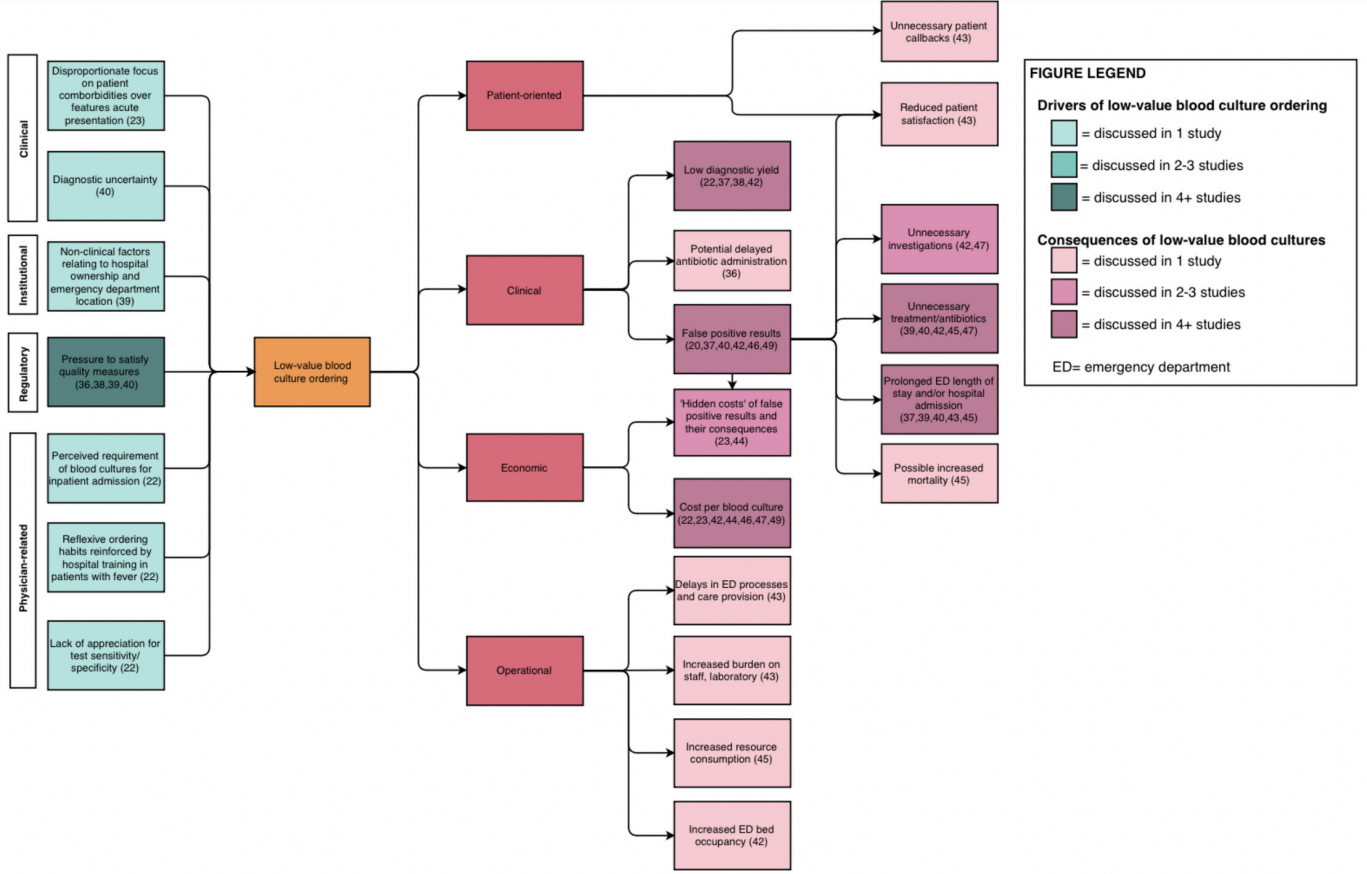
Conceptual map of the drivers and consequences of low‐value blood cultures in adult emergency department patients.

Seven studies [22, 23, 36, 40, 42, 43, 49] discussed the drivers of LVBC ordering. (Figure 2). The most frequently cited driver was perceived pressure to comply with performance measures, particularly the Centre of Medicaid Services (CMS) and Joint Commission (JCAHO) measure ‘PN‐3b’, mandating BC collection prior to antibiotic administration in all pneumonia patients [4, 28, 31, 32], and the 2018 Surviving Sepsis Campaign ‘1‐hour sepsis bundle’ [49]. Physician‐related factors included: reflexive ordering in response to fever, inadequate appreciation for test sensitivity/specificity, and ED physician belief that BCs were expected by inpatient teams [22]. Clinically, diagnostic uncertainty regarding unclear patient presentations [43] and disproportionate focus on comorbidities over acute presentation contributed to inappropriate ordering [23]. Makam et al. [42] also found that institutional factors such as hospital ownership and region were strongly linked to BC overuse.

### 3.9 Consequences of LVBCs

While none of the studies directly evaluated consequences of LVBCs, several described associated harms (Figure 2). Patient‐oriented harms were underreported, suggested by only one study which discussed unnecessary ED call‐backs and reduced patient satisfaction [47]. Clinical consequences were most frequently discussed, and included low true‐positive yield [22, 38, 40, 49], potential delays in appropriate antibiotic administration [36], and false‐positive results [20, 38, 41, 42, 43, 48, 49]. The downstream effects of false positives were also cited, including unnecessary investigations [37, 49], inappropriate antibiotics [42, 43, 46, 48, 49], prolonged hospitalisation or ED stays [38, 42, 43, 46, 47], and potentially increased mortality [46]. Operational harms involved delays in ED processes, increased burden on staff and laboratories [47], increased resource consumption [46] and ED bed occupancy [47].

Economic harms were described as the estimated financial impacts of unnecessary BCs, derived from per‐culture costs ranging from AUD $30 [22] to USD $145 [41]. This translated to estimated costs of LVBCs between AUD $18,000 [22] and $49,423 [37] annually, or up to USD $178,000 per 1000 patients [39]. Pawlowicz et al. [23] demonstrated savings of $36,000 USD per month following an intervention that reduced LVBCs. Additionally, two studies [23, 46] recognised ‘hidden costs’ pertaining to false positives, including increased length of stay and testing, though these were not quantified.

## 4 Discussion

This review synthesised existing evidence on the criteria defining LVBCs and the prevalence, drivers, and consequences of LVBC use in adult ED populations. Importantly, the concept of LVBCs reflects an interpretive synthesis rather than a definition used in primary studies. Findings should be understood within this context.

### 4.1 Variability in Measures of ‘Value’ and Estimated Prevalence of LVBCs

A key finding was the absence of explicit criteria defining what constitutes an LVBC. Instead, studies used various proxies such as limited impact on patient management, deviation from guideline recommendations, or risk stratification tool outputs.

Criteria for LVBCs varied widely, hence challenging research and quality improvement efforts. Many studies retrospectively classified LVBCs based on limited impact on patient management [38, 40]. While such outcome‐focused definitions offer measurable endpoints for research purposes, they risk underestimating the broader utility of BCs. ED clinicians decide to collect BCs in real time, without access to test results. Evaluations made using impacts on patient management as a marker of BC value are hence flawed, involving knowledge of culture results, confirmation bias, and circular reasoning. Narrow definitions of value overlook the role of negative BCs in confirming management and excluding the possibility of targeted therapies, within appropriate patient contexts. Retrospective assessments are further limited by documentation and hindsight bias, oversimplifying complex clinical reasoning [50]. ‘Limited impact on management’ alone should not define LVBCs.

Studies also explored the use of decision support tools that stratify bacteraemia risk and identify unnecessary BCs using data available at presentation [20, 23, 37, 39, 41, 45, 46, 47, 48] Most promisingly, the machine learning model developed by Schinkel et al. [46] was prospectively validated in a multicentre study, achieving an AUROC of 0.76 during prospective evaluation [46]. However, no tool studied was used consistently or involved in long term prospective adoption across a range of settings, limiting standardisation and generalisable feasibility.

### 4.2 Variability in Reported Prevalence of LVBCs

The range in prevalence of LVBCs (19% [47]–69% [20]) highlights the absence of standardised criteria. This likely reflects real‐world inconsistencies in BC ordering across institutions and geographic regions. Regardless, this demonstrates that a considerable proportion of BCs may represent low‐value care.

### 4.3 Clinical Contexts Associated With LVBCs

Common ED presentations such as CAP [23, 36, 38, 40, 42] and UTIs [45, 48] were frequently associated with LVBCs, reaffirming evidence that routine BCs in these conditions rarely provide benefits [4]. Notably, no included studies specifically examined uncomplicated cellulitis, despite a previous review [4] highlighting the low diagnostic yield of BCs in such conditions. This may reflect this review's eligibility criterion, limiting inclusion to studies with participants ≥ 18 years. Paolo et al. [44] also found limited utility for BCs in complicated cellulitis, challenging the assumption that higher‐risk populations automatically benefit from BCs, supporting patient‐specific decision‐making tools that look beyond broad clinical categories.

### 4.4 Drivers of LVBC Ordering

This review highlights a complex interplay of systemic mandates, cultural norms, and cognitive responses to uncertainty driving overuse (Figure 2). One frequently cited driver was the CMS/JCAHO core measure PN‐3b [51]. Although retired in 2014 [52], its 12‐year implementation underscores how policy can lag behind evolving evidence, reinforcing low‐value practices and normalising reflexive testing. Additionally, there is a lack of research exploring clinician motivations behind LVBC use, which will be necessary to address root causes.

### 4.5 Consequences of LVBC Use

The findings suggest LVBCs are associated with harms to patients and the health system based on associations such as false positives, unnecessary treatment, and increased length of stay, although no study formally investigated these.

The studies reflected an emphasis on clinical outcomes, with the lack of discussion surrounding patient‐centred outcomes such as physical discomfort, anxiety, and erosion of trust highlighting a critical knowledge gap. This also suggests a narrow, system‐focused framing of harm.

Economically, most analyses relied on projected rather than actual savings, limiting their utility regarding policy or budgeting. Only Pawlowicz et al. [23] reported actual cost reduction after improved use of BCs, demonstrating possible savings. Without prospective evaluation of these impacts, particularly from patient and economic perspectives, it will remain challenging to build a compelling case for change [53].

### 4.6 Limitations

The heterogeneity of studies limited the comparability of findings and strength of interpretive synthesis. Additionally, relevant studies may have been missed due to unrecognised limitations in the search strategy, database indexing, or exclusion of unpublished and grey literature. The majority of included studies involved retrospective designs, introducing risks of documentation bias and uncontrolled confounding factors, limiting study validity. Many studies were also conducted in single centres or within specific patient populations, limiting generalisability. Finally, the age‐based eligibility criterion applied to focus this review on adult patients inevitably excluded studies involving patients aged ≤ 17 years, although older adolescents are managed similarly to adults in EDs, possibly excluding clinically relevant data.

### 4.7 Implications for Practice and Future Research

This review highlights challenges in defining and managing LVBCs in adult EDs. Interventions are needed to address factors driving overuse. This will require clinician education, peer‐led reflection, and integration of real‐time decision support tools. Additionally, regulatory mandates must be revised to minimise avoidable harm and align with current evidence. Future research should focus on developing and validating standardised, prospectively applicable criteria to facilitate ongoing research efforts and allow for better identification of and reduction of inappropriate testing. Qualitative studies exploring both clinician decisions and patient perspectives will also be essential for designing de‐implementation strategies and understanding the consequences of LVBCs. Formal economic evaluations capturing direct and indirect costs of LVBCs are needed to support policy change. Additionally, systematic reviews surrounding strategies to support BC decision making, such as bacteraemia prediction tools, are needed to guide practice and identify the best tool(s) for optimal care.

## 5 Conclusion

LVBCs remain a common yet inconsistently defined challenge in adult emergency departments. This review has outlined the heterogeneous nature of current evidence, underscoring the need for clear, standardised, and prospectively applicable criteria to effectively identify and reduce LVBCs. Addressing this will require not only prospective decision support tools applicable to diverse ED settings, but also a cultural shift to target factors driving unnecessary testing. Future research should focus on validating such tools and characterising the patient‐centred and economic consequences of LVBCs, strengthening the foundation for improving BC use and optimising care for adult ED patients.

## Funding

The authors have nothing to report.

## Conflicts of Interest

The authors declare no conflicts of interest.

## Supporting information


**Appendix 1.** Preferred reporting items for systematic reviews and meta‐analyses extension for scoping reviews (PRISMA‐ScR) checklist.
**Appendix 2.** Inclusion and exclusion criteria.
**Appendix 3.** Database‐specific search strategies used for scoping review.
**Appendix 4.** Data charting tool used for scoping review.

## Data Availability

The data that support the findings of this study are available from the corresponding author upon reasonable request.

## References

[1] I. A. Scott and S. J. Duckett , “In Search of Professional Consensus in Defining and Reducing Low‐Value Care,” Medical Journal of Australia 203, no. 4 (2015): 179–181, 10.5694/mja14.01664. 26268286

[2] A. G. Elshaug , M. B. Rosenthal , J. N. Lavis , et al., “Levers for Addressing Medical Underuse and Overuse: Achieving High‐Value Health Care,” Lancet 390, no. 10090 (2017): 191–202, 10.1016/s0140-6736(16)32586-7. 28077228

[3] C. Rhee , R. Dantes , L. Epstein , et al., “Incidence and Trends of Sepsis in US Hospitals Using Clinical vs Claims Data, 2009–2014,” Journal of the American Medical Association 318, no. 13 (2017): 1241–1249, 10.1001/jama.2017.13836. 28903154 PMC5710396

[4] B. Long and A. Koyfman , “Best Clinical Practice: Blood Culture Utility in the Emergency Department,” Journal of Emergency Medicine 51, no. 5 (2016): 529–539, 10.1016/j.jemermed.2016.07.003. 27639424

[5] J. M. Klucher , K. Davis , M. Lakkad , J. T. Painter , and R. K. Dare , “Risk Factors and Clinical Outcomes Associated With Blood Culture Contamination,” Infection Control and Hospital Epidemiology 43, no. 3 (2022): 291–297, 10.1017/ice.2021.111. 33896442

[6] G. V. Doern , K. C. Carroll , D. J. Diekema , et al., “Practical Guidance for Clinical Microbiology Laboratories: A Comprehensive Update on the Problem of Blood Culture Contamination and a Discussion of Methods for Addressing the Problem,” Clinical Microbiology Reviews 33, no. 1 (2019): e00009–19, 10.1128/cmr.00009-19. 31666280 PMC6822992

[7] Australasian College for Emergency Medicine , “Recommendations: Do Not Order Blood Cultures in Patients Who Are Not Systemically Septic, Have a Clear Source of Infection, and in Whom a Direct Specimen for Culture Is Obtainable,” Choosing Wisely Australia, accessed August 15, 2025, https://www.choosingwisely.org.au/recommendations/acem3.

[8] E. C. Choi , Y. H. Chia , Y. Q. Koh , et al., “Appropriateness of Blood Culture: A Comparison of Practices Between the Emergency Department and General Wards,” Infection, Disease & Health 24, no. 1 (2019): 49–55, 10.1016/j.idh.2018.10.003. 30541693

[9] V. Fabre , A. M. Milstone , S. C. Keller , K. C. Carroll , and S. E. Cosgrove , “Prescribers' Knowledge, Attitudes and Perceptions About Blood Culturing Practices for Adult Hospitalized Patients: A Call for Action,” Infection Control and Hospital Epidemiology 39, no. 11 (2018): 1394–1396, 10.1017/ice.2018.224. 30226121

[10] K. Linsenmeyer , K. Gupta , J. M. Strymish , M. Dhanani , S. M. Brecher , and A. C. Breu , “Culture If Spikes? Indications and Yield of Blood Cultures in Hospitalized Medical Patients,” Journal of Hospital Medicine 11, no. 5 (2016): 336–340, 10.1002/jhm.2541. 26762577

[11] D. Bamber , N. Fahy , T. Coats , et al., “Factors Associated With Blood Culture Sampling for Adult Acute Care Hospital Patients With Suspected Severe Infection: A Scoping Review Using a Socioecological Framework,” JAC‐Antimicrobial Resistance 7, no. 2 (2025): dlaf043, 10.1093/jacamr/dlaf043. 40115169 PMC11924178

[12] D. W. Bates , L. Goldman , and T. H. Lee , “Contaminant Blood Cultures and Resource Utilization. The True Consequences of False‐Positive Results,” Journal of the American Medical Association 265, no. 3 (1991): 365–369. 1984535

[13] K. K. Hall and J. A. Lyman , “Updated Review of Blood Culture Contamination,” Clinical Microbiology Reviews 19, no. 4 (2006): 788–802, 10.1128/cmr.00062-05. 17041144 PMC1592696

[14] R. M. Gander , L. Byrd , M. DeCrescenzo , S. Hirany , M. Bowen , and J. Baughman , “Impact of Blood Cultures Drawn by Phlebotomy on Contamination Rates and Health Care Costs in a Hospital Emergency Department,” Journal of Clinical Microbiology 47, no. 4 (2009): 1021–1024, 10.1128/jcm.02162-08. 19171686 PMC2668314

[15] Y. F. van der Heijden , G. Miller , P. W. Wright , B. E. Shepherd , T. L. Daniels , and T. R. Talbot , “Clinical Impact of Blood Cultures Contaminated With Coagulase‐Negative Staphylococci at an Academic Medical Center,” Infection Control and Hospital Epidemiology 32, no. 6 (2011): 623–625, 10.1086/660096. 21558778

[16] “PRISMA Extension for Scoping Reviews (PRISMA‐ScR): Checklist and Explanation,” Annals of Internal Medicine 169, no. 7 (2018): 467–473, 10.7326/m18-0850m30178033. 30178033

[17] M. D. J. Peters , C. Godfrey , P. McInerney , Z. Munn , A. C. Tricco , and H. Khalil , “Chapter 11: Scoping Reviews,” in JBI Manual for Evidence Synthesis, ed. E. Aromataris , C. Lockwood , K. Porritt , B. Pilla , and Z. Jordan (JBI, 2024).

[18] “EndNote. Version 21,” Clarivate, (2023).

[19] Z. Munn , E. Aromataris , C. Tufanaru , et al., “The Development of Software to Support Multiple Systematic Review Types: The Joanna Briggs Institute System for the Unified Management, Assessment and Review of Information (JBI SUMARI),” JBI Evidence Implementation 17, no. 1 (2019): 36–43, 10.1097/xeb.0000000000000152. 30239357

[20] A. W. Boerman , M. Schinkel , L. Meijerink , et al., “Using Machine Learning to Predict Blood Culture Outcomes in the Emergency Department: A Single‐Centre, Retrospective, Observational Study,” BMJ Open 12, no. 1 (2022): e053332, 10.1136/bmjopen-2021-053332. PMC8728456 34983764

[21] B. P. Ehrenstein , T. Jarry , H. J. Linde , J. Schölmerich , and T. Glück , “Low Rate of Clinical Consequences Derived From Results of Blood Cultures Obtained in an Internal Medicine Emergency Department,” Infection 33, no. 5–6 (2005): 314–319, 10.1007/s15010-005-5065-5. 16258860

[22] A. M. Kelly , “Clinical Impact of Blood Cultures Taken in the Emergency Department,” Emergency Medicine Journal 15, no. 4 (1998): 254–256, 10.1136/emj.15.4.254. PMC1343139 9681310

[23] A. Pawlowicz , C. Holland , B. Zou , T. Payton , J. A. Tyndall , and B. Allen , “Implementation of an Evidence‐ Based Algorithm Reduces Blood Culture Overuse in an Adult Emergency Department,” General Internal Medicine and Clinical Innovation 1, no. 2 (2016): 20–25, 10.15761/GIMCI.1000108.

[24] L. N. Ko , A. C. Garza‐Mayers , J. St John , et al., “Clinical Usefulness of Imaging and Blood Cultures in Cellulitis Evaluation,” JAMA Internal Medicine 178, no. 7 (2018): 994–996, 10.1001/jamainternmed.2018.0625. 29610842 PMC6145713

[25] B. Kim , K. Kim , J. Lee , et al., “Impact of Bacteremia Prediction Rule in CAP: Before and After Study,” American Journal of Emergency Medicine 36, no. 5 (2018): 758–762, 10.1016/j.ajem.2017.10.005. 28988847 PMC7127687

[26] R. S. Kamath , D. Sudhakar , J. G. Gardner , V. Hemmige , H. Safar , and D. M. Musher , “Guidelines vs Actual Management of Skin and Soft Tissue Infections in the Emergency Department,” Open Forum Infectious Diseases 5, no. 1 (2018): ofx188, 10.1093/ofid/ofx188. 29354655 PMC5767964

[27] N. Howie , J. F. Gerstenmaier , and P. T. Munro , “Do Peripheral Blood Cultures Taken in the Emergency Department Influence Clinical Management?,” Emergency Medicine Journal 24, no. 3 (2007): 213–214, 10.1136/emj.2006.039875. 17351231 PMC2660034

[28] A. Esposito , M. E. Silverman , F. Diaz , F. Fiesseler , G. Magnes , and D. Salo , “Sepsis Core Measures – Are They Worth the Cost?,” Journal of Emergency Medicine 55, no. 6 (2018): 751–757, 10.1016/j.jemermed.2018.07.033. 30253948

[29] M. J. Page , J. E. McKenzie , P. M. Bossuyt , et al., “The PRISMA 2020 Statement: An Updated Guideline for Reporting Systematic Reviews,” BMJ 372 (2021): n71, 10.1136/bmj.n71. 33782057 PMC8005924

[30] “Microsoft Excel for Mac. Version 16.99.1,” Microsoft, (2025).

[31] Z. Munn , T. Barker , S. Moola , et al., “Methodological Quality of Case Series Studies: An Introduction to the JBI Critical Appraisal Tool,” JBI Evidence Synthesis 18, no. 10 (2020): 2127–2133. 33038125 10.11124/JBISRIR-D-19-00099

[32] J. M. Campbell , M. Klugar , S. Ding , et al., “Chapter 9: Diagnostic Test Accuracy Systematic Reviews,” in JBI Manual for Evidence Synthesis, ed. E. M. Z. Aromataris (JBI, 2020).

[33] T. H. Barker , N. Habibi , E. Aromataris , et al., “The Revised JBI Critical Appraisal Tool for the Assessment of Risk of Bias Quasi‐Experimental Studies,” JBI Evidence Synthesis 22, no. 3 (2024): 378–388. 38287725 10.11124/JBIES-23-00268

[34] S. Moola , Z. Munn , C. Tufanaru , et al., “Chapter 7: Systematic Reviews of Etiology and Risk,” in JBI Manual for Evidence Synthesis, ed. E. M. Z. Aromataris (JBI, 2020).

[35] A. George , L. Lombardo , S. Ajwani , et al., “The Characteristics and Effectiveness of Oral Healthcare Education Interventions for Stroke Clinicians: A Scoping Review,” Journal of Clinical Nursing 34, no. 9 (2025): 3473–3488, 10.1111/jocn.17795. 40296502 PMC12340753

[36] R. S. Benenson , A. M. Kepner , I. D. N. Pyle , and S. Cavanaugh , “Selective Use of Blood Cultures in Emergency Department Pneumonia Patients,” Journal of Emergency Medicine 33, no. 1 (2007): 1–8, 10.1016/j.jemermed.2006.12.034. 17630066

[37] J. D. Brown , S. Chapman , and P. E. Ferguson , “Blood Cultures and Bacteraemia in an Australian Emergency Department: Evaluating a Predictive Rule to Guide Collection and Their Clinical Impact,” Emergency Medicine Australasia 29, no. 1 (2017): 56–62, 10.1111/1742-6723.12696. 27758065

[38] J. Corbo , B. Friedman , P. Bijur , and E. J. Gallagher , “Limited Usefulness of Initial Blood Cultures in Community Acquired Pneumonia,” Emergency Medicine Journal 21, no. 4 (2004): 446–448, https://www.scopus.com/inward/record.uri?eid=2‐s2.0‐3242756686&partnerID=40&md5=736b29696cabb832afacff4dd20f627c. 15208227 PMC1726373

[39] A. G. Kaal , S. Meziyerh , N. van Burgel , et al., “Procalcitonin for Safe Reduction of Unnecessary Blood Cultures in the Emergency Department: Development and Validation of a Prediction Model,” Journal of Infection 89, no. 4 (2024): 106251, 10.1016/j.jinf.2024.106251. 39182652

[40] M. Kennedy , D. W. Bates , S. B. Wright , R. Ruiz , R. E. Wolfe , and N. I. Shapiro , “Do Emergency Department Blood Cultures Change Practice in Patients With Pneumonia?,” Annals of Emergency Medicine 46, no. 5 (2005): 393–400, 10.1016/j.annemergmed.2005.05.025. 16271664

[41] S. Laukemann , N. Kasper , P. Kulkarni , et al., “Can We Reduce Negative Blood Cultures With Clinical Scores and Blood Markers? Results From an Observational Cohort Study,” Medicine (United States) 94, no. 49 (2015): e2264. 10.1097/MD.0000000000002264 PMC5008518 26656373

[42] A. N. Makam , A. D. Auerbach , and M. A. Steinman , “Blood Culture Use in the Emergency Department in Patients Hospitalized for Community‐Acquired Pneumonia,” JAMA Internal Medicine 174, no. 5 (2014): 803–806, 10.1001/jamainternmed.2013.13808. 24614986 PMC4123670

[43] A. N. Makam , A. D. Auerbach , and M. A. Steinman , “Blood Culture Use in the Emergency Department Inpatients Hospitalised With Respiratory Symtpoms due to Non‐Pneumonia Illness,” Journal of Hospital Medicine 9, no. 8 (2015): 521–524, 10.1002/jhm.2205. PMC4128469 24753399

[44] W. F. Paolo , A. R. Poreda , W. Grant , and W. S. Scordino , “Blood Cutlure Results Do Not Affect Treatment in Complicated Cellulitis,” Journal of Emergency Medicine 45, no. 2 (2013): 163–167, 10.1016/j.jemermed.2013.01.016. 23588078

[45] K. Rothe , C. D. Spinner , B. Waschulzik , et al., “A Diagnostic Algorithm for Detection of Urinary Tract Infections in Hospitalized Patients With Bacteriuria: The “Triple F” Approach Supported by Procalcitonin and Paired Blood and Urine Cultures,” PLoS One 15 (2020): e0240981, 10.1371/journal.pone.0222545. 33091046 PMC7580978

[46] M. Schinkel , A. W. Boerman , F. C. Bennis , et al., “Diagnostic Stewardship for Blood Cultures in the Emergency Department: A Multicenter Validation and Prospective Evaluation of a Machine Learning Prediction Tool,” eBioMedicine 82 (2022): 104176, 10.1016/j.ebiom.2022.104176. 35853298 PMC9294655

[47] R. Theophanous , J. Ramos , A. R. Calland , et al., “Blood Culture Algorithm Implementation in Emergency Department Patients as a Diagnostic Stewardship Intervention,” American Journal of Infection Control 52, no. 9 (2024): 985–991, 10.1016/j.ajic.2024.04.198. 38719159

[48] C. van Nieuwkoop , T. N. Bonten , J. W. Van't Wout , et al., “Procalcitonin Reflects Bacteremia and Bacterial Load in Urosepsis Syndrome: A Prospective Observational Study,” Critical Care 14, no. 6 (2010): R206, 10.1186/cc9328. 21083886 PMC3220019

[49] T. Zitek , M. Bourne , J. Raber , A. Shir , and B. Ryabtsev , “Blood Culture Results and Overtreatment Associated With the Use of a 1‐Hour Sepsis Bundle,” Journal of Emergency Medicine 59, no. 5 (2020): 629–636. 32741577 10.1016/j.jemermed.2020.06.055

[50] R. L. Wears and C. P. Nemeth , “Replacing Hindsight With Insight: Toward Better Understanding of Diagnostic Failures,” Annals of Emergency Medicine 49, no. 2 (2007): 206–209, 10.1016/j.annemergmed.2006.08.027. 17083994

[51] The Joint Commision , “Blood Cultures Performed in the Emergency Department Prior to Initial Antibiotic Received in Hospital (PN‐3b),” in Specifications Manual for Joint Commission National Quality Core Measures (2010A1) (Joint Commision, 2017).

[52] T. Swenson and M. Casey , MBQIP Quality bMeasure Trends, 2011–2016 (FMT Data Summary Report #20) (Flex Monitoring Team, 2016).

[53] T. Hoomans and J. L. Severens , “Economic Evaluation of Implementation Strategies in Health Care,” Implementation Science 9, no. 1 (2014): 168, 10.1186/s13012-014-0168-y. 25518730 PMC4279808

