# Supplementary material for: Low‐Value Blood Culture Use in Adult Emergency Department Patients: A Scoping Review
Source: Emerg Med Australas. 2026 Jun 28;38(3):e70299. doi: 10.1111/1742-6723.70299 (PMC13310798; doi:10.1111/1742-6723.70299)
Supplement: Supplementary file 1 — Appendix 1. Preferred reporting items for systematic reviews and meta‐analyses extension for scoping reviews (PRISMA‐ScR) checklist. Appendix 2. Inclusion and exclusion criteria. Appendix 3. Database‐specific search strategies used for scoping review. Appendix 4. Data charting tool used for scoping review. [file EMM-38-0-s001.docx]

**Appendix 1.** Preferred Reporting Items for Systematic reviews and Meta-Analyses extension for Scoping Reviews (PRISMA-ScR) Checklist

| **SECTION** | **ITEM** | **PRISMA-ScR CHECKLIST ITEM** | **REPORTED ON PAGE #** |
| --- | --- | --- | --- |
| **TITLE** | | | |
| Title | 1 | Identify the report as a scoping review. | 1 |
| **ABSTRACT** | | | |
| Structured summary | 2 | Provide a structured summary that includes (as applicable): background, objectives, eligibility criteria, sources of evidence, charting methods, results, and conclusions that relate to the review questions and objectives. | 1-2 |
| **INTRODUCTION** | | | |
| Rationale | 3 | Describe the rationale for the review in the context of what is already known. Explain why the review questions/objectives lend themselves to a scoping review approach. | 3 |
| Objectives | 4 | Provide an explicit statement of the questions and objectives being addressed with reference to their key elements (e.g., population or participants, concepts, and context) or other relevant key elements used to conceptualize the review questions and/or objectives. | 4 |
| **METHODS** | | | |
| Protocol and registration | 5 | Indicate whether a review protocol exists; state if and where it can be accessed (e.g., a Web address); and if available, provide registration information, including the registration number. | 4 |
| Eligibility criteria | 6 | Specify characteristics of the sources of evidence used as eligibility criteria (e.g., years considered, language, and publication status), and provide a rationale. | 4 |
| Information sources* | 7 | Describe all information sources in the search (e.g., databases with dates of coverage and contact with authors to identify additional sources), as well as the date the most recent search was executed. | 5 |
| Search | 8 | Present the full electronic search strategy for at least 1 database, including any limits used, such that it could be repeated. | 5 |
| Selection of sources of evidence† | 9 | State the process for selecting sources of evidence (i.e., screening and eligibility) included in the scoping review. | 5 |
| Data charting process‡ | 10 | Describe the methods of charting data from the included sources of evidence (e.g., calibrated forms or forms that have been tested by the team before their use, and whether data charting was done independently or in duplicate) and any processes for obtaining and confirming data from investigators. | 6 |
| Data items | 11 | List and define all variables for which data were sought and any assumptions and simplifications made. | 6 |
| Critical appraisal of individual sources of evidence§ | 12 | If done, provide a rationale for conducting a critical appraisal of included sources of evidence; describe the methods used and how this information was used in any data synthesis (if appropriate). | 6 |

**Appendix 1.** Preferred Reporting Items for Systematic reviews and Meta-Analyses extension for Scoping Reviews (PRISMA-ScR) Checklist (continued)

| **SECTION** | **ITEM** | **PRISMA-ScR CHECKLIST ITEM** | **REPORTED ON PAGE #** |
| --- | --- | --- | --- |
| Synthesis of results | 13 | Describe the methods of handling and summarizing the data that were charted. | 6 |
| **RESULTS** | | | |
| Selection of sources of evidence | 14 | Give numbers of sources of evidence screened, assessed for eligibility, and included in the review, with reasons for exclusions at each stage, ideally using a flow diagram. | 6-7 |
| Characteristics of sources of evidence | 15 | For each source of evidence, present characteristics for which data were charted and provide the citations. | 7 |
| Critical appraisal within sources of evidence | 16 | If done, present data on critical appraisal of included sources of evidence (see item 12). | 7-8 |
| Results of individual sources of evidence | 17 | For each included source of evidence, present the relevant data that were charted that relate to the review questions and objectives. | 8-11 |
| Synthesis of results | 18 | Summarize and/or present the charting results as they relate to the review questions and objectives. | 8-11 |
| **DISCUSSION** | | | |
| Summary of evidence | 19 | Summarize the main results (including an overview of concepts, themes, and types of evidence available), link to the review questions and objectives, and consider the relevance to key groups. | 11-14 |
| Limitations | 20 | Discuss the limitations of the scoping review process. | 14 |
| Conclusions | 21 | Provide a general interpretation of the results with respect to the review questions and objectives, as well as potential implications and/or next steps. | 15 |
| **FUNDING** | | | |
| Funding | 22 | Describe sources of funding for the included sources of evidence, as well as sources of funding for the scoping review. Describe the role of the funders of the scoping review. | 15 |

JBI = Joanna Briggs Institute; PRISMA-ScR = Preferred Reporting Items for Systematic reviews and Meta-Analyses extension for Scoping Reviews.

* Where *sources of evidence* (see second footnote) are compiled from, such as bibliographic databases, social media platforms, and Web sites.

† A more inclusive/heterogeneous term used to account for the different types of evidence or data sources (e.g., quantitative and/or qualitative research, expert opinion, and policy documents) that may be eligible in a scoping review as opposed to only studies. This is not to be confused with *information sources* (see first footnote).

‡ The frameworks by Arksey and O’Malley (6) and Levac and colleagues (7) and the JBI guidance (4, 5) refer to the process of data extraction in a scoping review as data charting*.*

§ The process of systematically examining research evidence to assess its validity, results, and relevance before using it to inform a decision. This term is used for items 12 and 19 instead of "risk of bias" (which is more applicable to systematic reviews of interventions) to include and acknowledge the various sources of evidence that may be used in a scoping review (e.g., quantitative and/or qualitative research, expert opinion, and policy document).

*From:* Tricco AC, Lillie E, Zarin W, O'Brien KK, Colquhoun H, Levac D, et al. PRISMA Extension for Scoping Reviews (PRISMAScR): Checklist and Explanation. Ann Intern Med. 2018;169:467–473. [doi: 10.7326/M18-0850](http://annals.org/aim/fullarticle/2700389/prisma-extension-scoping-reviews-prisma-scr-checklist-explanation).

**Appendix 2.** Inclusion and Exclusion Criteria

| Domain | Inclusion Criteria | Exclusion Criteria |
| --- | --- | --- |
| Population | Human studies involving adults >18 years ^a^ | Animal studies, paediatric studies |
| Concept | Studies exploring the low-value use of BCs, including those that assess their clinical utility and conclude they are of limited value (e.g., inappropriate, excessive, low-yield, or not influencing patient management). | Studies that do not address LVBC use  Studies which focus solely on BC collection technique, contamination prevention and/or laboratory methods |
| Context | Emergency department (BCs must be those ordered in the emergency department) | Non-ED settings (e.g. Intensive care units) |
| Study Design | Primary studies including quantitative, qualitative and mixed methods studies | Case reports, case series.  Non-primary sources such as reviews, protocols, perspectives, comment, opinions, editorials, letters to editors, news articles, books, chapters, policies, guidelines |
| Risk of bias | Included regardless of risk of bias | - |
| Sample Size | Included regardless of sample size | - |
| Language | Studies included regardless of language of publication | Studies that cannot be adequately translated into English, even after reasonable attempts |

**a** Age criterion included to ensure consistency in clinical context, diagnostic protocols, and management practices, which can differ significantly between paediatric and adult populations. Including paediatric or mixed-age cohorts may have introduced additional heterogeneity, potentially limiting the interpretability and applicability of the findings. Where the age of the study population was unclear, the corresponding author was contacted to confirm inclusion of only adult (≥18 years) individuals. Studies were excluded if this could not be confirmed and author contact was unsuccessful. Studies specifying adult populations without explicit age details were included, assuming adults were aged ≥18 years as per country norms.

**Appendix 3.** Database-specific search strategies used for scoping review

| Database: MEDLINE | |  |
| --- | --- | --- |
| Search | **Query** | **Number of records** |
| #4 | #1 AND #2 AND #3 | 168 |
| #3 | Low-value care/ or Health services misuse/ or Medical overuse/ or Unnecessary procedures/ or medical errors/ or overdiagnosis/ or overtreatment/ or "low-value" or "low value" or "excessive" or "low yield" or "low-yield" or "inappropriate" or "unnecessary" or overuse* or misuse* or "over utilization" or "over-utilization" or "overutilization" or "overutilized" or "overutilised" or "over utilisation" or "over-utilisation" or "overutlisation" or "unwanted" or "unwarranted" or "unjustified" or "over testing" or "overdiagnosis" or "over diagnosis" or "over-diagnosis overtesting" or "over-testing" or "suboptimal" or "sub-optimal" or "poor quality" or "poor-quality" or "low utility" or "limited utility" or "poor utility" or "rarely useful" or "rarely positive" or "overtreatment" or "over-treatment" or "over treatment" | 572 443 |
| #2 | Emergency medical services/ or emergency room visits/ or exp emergency service, hospital/ or emergency medicine/ or "A and E Department" or "A and E Service" or "A and E Services" or "A and E Departments" or "Accident and Emergency Service" or "Accident and Emergency Services" or "A and E unit" or "A and E units" or "A-E department" or "A-E departments" or "A-E unit" or "A-E unit” or "A-E ward" or "A-E wards" or "accident and emergency ward" or "accident and emergency wards" or "AE department" or "AE departments" or "AE unit" or "AE units" or "AE ward" or "AE wards" or "AED" or "AEDs" or "casualty department" or "casualty departments" or "casualty room" or "casualty rooms" or "Casualty unit" or "casualty units" or "emergency unit" or "emergency units" or "Accident and Emergency Department" or "accident and emergency departments" or "Casualty Department" or "Casualty Departments" or "Emergency Department" or "Emergency Departments" or "Emergency Room" or "Emergency Rooms" or "emergency service" or "Emergency Services" or "Emergicenter" or "emergicenter" or "Emergicenters" or "emergicentres" or "Emergency Hospital Service" or "Emergency Hospital Services" or "Hospital Emergency Services" or "Hospital emergency service" or "Emergency Ward" or "Emergency Wards" or "emergency unit" or "Emergency Units" or "Hospital Emergency Room" or "Hospital Emergency Rooms" or "Emergency Outpatient Unit" or "Emergency Outpatient Units" or "Trauma Center" or "Trauma Centers" or "Trauma Centre" or "Trauma centres" or "Trauma Units" or "Trauma Unit" or "Emergency Health Service" or "Emergency Health Services" or "Emergency Medical Service" or "Emergency Medical Services" or "Medical Emergency Service" or "Medical Emergency Services" or "Emergency Patient" or "Emergency Patients" or "Trauma Patient" or "Trauma Patients" or "ER Physician" or "ER Physicians" or "Emergency Doctor" or "emergency doctors" or "Emergency Physician" or "Emergency Physicians" or "Emergency Room Physician" or "Emergency Room Physicians" or "Emergency Medicine" or "Emergency Care" or "emergency clinician" or "emergency clinicians" or "emergency specialist" or "emergency specialists" or "emergenotologist*" or "ED" or "EDs" | 436 781 |
| #1 | Blood cultures/ or blood specimen collection/ or "blood culture" or "blood cultures” | 33 348 |

**Appendix 3.** Database-specific search strategies used for scoping review (continued)

| Database: CINAHL | |  |
| --- | --- | --- |
| Search | **Query** | **Number of records** |
| #4 | #1 AND #2 AND #3 | 97 |
| #3 | (MH “Low-value care”) OR (MH “Health Services Misuse”) OR (MH “Overtreatment”) OR (MH “Unnecessary Procedures”) OR (MH “overdiagnosis”) OR “low-value” OR “low value” OR “excessive” OR “low yield” OR “low-yield” OR “inappropriate” OR “unnecessary” OR “overuse*” OR “misuse*” OR “over utilization” OR “over-utilization” OR “overutiliz*” OR “over utilisation” OR “over-utilisation” OR “overutilis*” OR “unwanted” OR “unwarranted” OR “unjustified” OR “over testing” OR “overtesting” OR “over-testing” OR “over diagnosis” OR “overdiagnosis” OR “over-diagnosis” OR “suboptimal” OR “sub-optimal” OR “poor quality” OR “poor-quality” OR “low utility” OR “limited utility” OR “poor utility” OR “not useful” OR “not helpful” OR “rarely useful” OR “rarely positive” OR “overtreatment” OR “over treatment” OR “over-treatment” | 308 322 |
| #2 | (MH “Emergency service+”) or (MH “Emergency Medical Services”) or (MH “Emergency patients”) or (MH “Physicians, Emergency”) or (MH “Emergency medicine”) or (MH “Emergency care”) OR "ae unit*" OR "ae ward*" OR "aed*" OR "ae department" OR "a and e department*" OR "a and e service*" OR "a and e unit*" OR "a-e department*" OR "a-e unit*" OR "a-e ward" OR "accident and emergency service*" OR "accident and emergency ward*" OR "accident and emergency department*" OR "emergency ward*" OR "emergency room*" OR "casualty room*" OR "casualty department*" OR "casualty unit*" OR "emergicenter*" OR "emergicenter*" OR "emergency service*" OR "emergency room*" OR "emergency department*" OR "emergency hospital service*" OR "hospital emergency service*" OR "hospital emergency room*" OR "emergency outpatient unit*" OR "emergency medical service*" OR "emergency outpatient clinic*" OR "emergency care" OR "emergency medicine" OR "emergency visit*" OR "emergicenter*" OR "emergicenter*" OR "emergency health service*" OR "emergency medical service*" OR "medical emergency service*" OR "emergency unit*" OR "emergency clinician*" OR "emergency specialist*" OR "emergentologist*" OR "emergency room physician*" OR "emergency doctor*" OR "emergency medicine" OR "emergency care" OR "er physician*" OR "trauma patient*" OR "emergency patient*" OR "ed" OR "eds" OR "trauma centre*" OR "trauma center*" OR "trauma unit*" | 224 538 |
| #1 | (MH “Blood culture”) or “Blood culture” or “Blood cultures" | 6 223 |

**Appendix 3.** Database-specific search strategies used for scoping review (continued)

| Database: EMBASE | |  |
| --- | --- | --- |
| Search | **Query** | **Number of records** |
| #4 | #1 AND #2 AND #3 | 592 |
| #3 | low-value care'/exp OR 'low-value care' OR 'unnecessary procedure'/exp OR 'unnecessary procedure' OR 'medical overuse'/exp OR 'medical overuse' OR 'health service misuse'/exp OR 'health service misuse' OR 'low-value' OR 'low value' OR 'excessive' OR 'low yield' OR 'low-yield' OR 'inappropriate' OR 'unnecessary' OR 'overuse*' OR 'misuse*' OR 'over utilization' OR 'over-utilization' OR 'overutilization' OR 'overutilized' OR 'overutilised' OR 'over utilisation' OR 'over-utilisation' OR 'overutlisation' OR 'unwanted' OR 'unwarranted' OR 'unjustified' OR 'over testing' OR 'overdiagnosis'/exp OR 'overdiagnosis' OR 'over diagnosis'/exp OR 'over diagnosis' OR 'over-diagnosis'/exp OR 'over-diagnosis' OR 'overtesting'/exp OR 'overtesting' OR 'suboptimal' OR 'sub-optimal' OR 'poor quality' OR 'poor-quality' OR 'low utility' OR 'limited utility' OR 'poor utility' OR 'rarely useful' OR 'rarely positive' OR 'overtreatment'/exp OR 'overtreatment' OR 'over-treatment' OR 'over treatment' | 798 668 |
| #2 | emergency outpatient clinic'/exp OR 'emergency outpatient clinic' OR 'emergency medicine'/exp OR 'emergency medicine' OR 'emergency patient'/exp OR 'emergency patient' OR 'evidence based emergency medicine'/exp OR 'evidence based emergency medicine' OR 'emergency visit'/exp OR 'emergency visit' OR 'a and e department*' OR 'a and e service*' OR 'accident and emergency service*' OR 'a and e unit'/exp OR 'a and e unit' OR 'a and e units' OR 'a-e department*' OR 'a-e unit'/exp OR 'a-e unit' OR 'a-e ward'/exp OR 'a-e ward' OR 'a-e wards' OR 'accident and emergency ward'/exp OR 'accident and emergency ward' OR 'accident and emergency wards' OR 'ae department*' OR 'ae unit'/exp OR 'ae unit' OR 'ae units' OR 'ae ward'/exp OR 'ae ward' OR 'ae wards' OR 'aed' OR 'aeds' OR 'casualty room'/exp OR 'casualty room' OR 'casualty rooms' OR 'casualty unit'/exp OR 'casualty unit' OR 'casualty units' OR 'accident and emergency department*' OR 'casualty department*' OR 'emergency department*' OR 'emergency room'/exp OR 'emergency room' OR 'emergency rooms' OR 'emergency service*' OR 'emergicentre' OR 'emergicenter*' OR 'emergency hospital service*' OR 'hospital emergency service*' OR 'emergency ward'/exp OR 'emergency ward' OR 'emergency wards' OR 'emergency unit'/exp OR 'emergency unit' OR 'emergency units' OR 'hospital emergency room' OR 'hospital emergency rooms' OR 'emergency outpatient unit'/exp OR 'emergency outpatient unit' OR 'emergency outpatient units' OR 'trauma center*' OR 'trauma centre*' OR 'trauma unit*' OR 'emergency health service*' OR 'emergency medical service*' OR 'medical emergency service*' OR 'emergency patient*' OR 'trauma patient*' OR 'er physician*' OR 'emergency doctor*' OR 'emergency physician*' OR 'emergency room physician*' OR 'emergency care'/exp OR 'emergency care' OR 'emergency clinician*' OR 'emergency specialist*' OR 'emergentologist*' OR 'ed' OR 'eds'/exp OR 'eds' | 1 180 735 |
| #1 | blood culture'/ OR 'blood culture' OR 'blood cultures' | 88 653 |

**Appendix 3.** Database-specific search strategies used for scoping review (continued)

| Database: EMCARE | |  |
| --- | --- | --- |
| Search | **Query** | **Number of records** |
| #4 | #1 AND #2 AND #3 | 144 |
| #3 | low-value care/ OR unnecessary procedure/ OR medical overuse/ OR overtreatment/ OR overtesting/ OR overtreatment/ OR health service misuse/ OR “low-value” OR “low value” OR “excessive” OR “low yield” OR “low-yield” OR “inappropriate” OR “unnecessary” OR “overuse*” OR “misuse*” OR “over utilization” OR “over-utilization” OR “overutiliz*” OR “over utilisation” OR “over-utilisation” OR “overutilis*” OR “unwanted” OR “unwarranted” OR “unjustified” OR “over testing” OR “overtesting” OR “over-testing” OR “over diagnosis” OR “overdiagnosis” OR “over-diagnosis” OR “suboptimal” OR “sub-optimal” OR “poor quality” OR “poor-quality” OR “low utility” OR “limited utility” OR “poor utility” OR “not useful” OR “not helpful” OR “rarely useful” OR “rarely positive” OR “overtreatment” OR “over treatment” OR “over-treatment” | 203 626 |
| #2 | Emergency ward/ OR emergency health service/ OR hospital emergency service/ OR emergency physician/ OR emergency outpatient clinic/ OR emergency medicine/ OR emergency patient/ OR evidence based emergency medicine/ OR exp emergency visit/ OR "ae unit*" OR "ae ward*" OR "aed*" OR "ae department" OR "a and e department*" OR "a and e service*" OR "a and e unit*" OR "a-e department*" OR "a-e unit*" OR "a-e ward" OR "accident and emergency service*" OR "accident and emergency ward*" OR "accident and emergency department*" OR "emergency ward*" OR "emergency room*" OR "casualty room*" OR "casualty department*" OR "casualty unit*" OR "emergicenter*" OR "emergicenter*" OR "emergency service*" OR "emergency room*" OR "emergency department*" OR "emergency hospital service*" OR "hospital emergency service*" OR "hospital emergency room*" OR "emergency outpatient unit*" OR "emergency medical service*" OR "emergency outpatient clinic*" OR "emergency care" OR "emergency medicine" OR "emergency visit*" OR "emergicenter*" OR "emergicenter*" OR "emergency health service*" OR "emergency medical service*" OR "medical emergency service*" OR "emergency unit*" OR "emergency clinician*" OR "emergency specialist*" OR "emergentologist*" OR "emergency room physician*" OR "emergency doctor*" OR "emergency medicine" OR "emergency care" OR "er physician*" OR "trauma patient*" OR "emergency patient*" OR "ed" OR "eds" OR "trauma centre*" OR "trauma center*" OR "trauma unit*" | 272 184 |
| #1 | Blood culture/ OR "blood culture" OR "blood cultures" | 19 951 |

**Appendix 3.** Database-specific search strategies used for scoping review (continued)

| Database: Scopus | |  |
| --- | --- | --- |
| Search | **Query** | **Number of records** |
| #4 | #1 AND #2 AND #3 | 274 |
| #3 | “low-value” OR “low value” OR “excessive” OR “low yield” OR “low-yield” OR “inappropriate” OR “unnecessary” OR “overuse*” OR “misuse*” OR “over utilization” OR “over-utilization” OR “overutiliz*” OR “over utilisation” OR “over-utilisation” OR “overutilis*” OR “unwanted” OR “unwarranted” OR “unjustified” OR “over testing” OR “overtesting” OR “over-testing” OR “over diagnosis” OR “overdiagnosis” OR “over-diagnosis” OR “suboptimal” OR “sub-optimal” OR “poor quality” OR “poor-quality” OR “low utility” OR “limited utility” OR “poor utility” OR “not useful” OR “not helpful” OR “rarely useful” OR “rarely positive” OR “overtreatment” OR “over treatment” OR “over-treatment” | 1 207 446 |
| #2 | "ae unit*" OR "ae ward*" OR "aed*" OR "ae department" OR "a and e department*" OR "a and e service*" OR "a and e unit*" OR "a-e department*" OR "a-e unit*" OR "a-e ward" OR "accident and emergency service*" OR "accident and emergency ward*" OR "accident and emergency department*" OR "emergency ward*" OR "emergency room*" OR "casualty room*" OR "casualty department*" OR "casualty unit*" OR "emergicenter*" OR "emergicenter*" OR "emergency service*" OR "emergency room*" OR "emergency department*" OR "emergency hospital service*" OR "hospital emergency service*" OR "hospital emergency room*" OR "emergency outpatient unit*" OR "emergency medical service*" OR "emergency outpatient clinic*" OR "emergency care" OR "emergency medicine" OR "emergency visit*" OR "emergicenter*" OR "emergicenter*" OR "emergency health service*" OR "emergency medical service*" OR "medical emergency service*" OR "emergency unit*" OR "emergency clinician*" OR "emergency specialist*" OR "emergentologist*" OR "emergency room physician*" OR "emergency doctor*" OR "emergency medicine" OR "emergency care" OR "er physician*" OR "trauma patient*" OR "emergency patient*" OR "ed" OR "eds" OR "trauma centre*" OR "trauma center*" OR "trauma unit*" | 787 310 |
| #1 | “blood culture” OR “blood cultures” | 67 976 |

**Appendix 3.** Database-specific search strategies used for scoping review (continued)

| Database: Web of Science | |  |
| --- | --- | --- |
| Search | **Query** | **Number of records** |
| #4 | #1 AND #2 AND #3 | 167 |
| #3 | “low-value” OR “low value” OR “excessive” OR “low yield” OR “low-yield” OR “inappropriate” OR “unnecessary” OR “overuse*” OR “misuse*” OR “over utilization” OR “over-utilization” OR “overutiliz*” OR “over utilisation” OR “over-utilisation” OR “overutilis*” OR “unwanted” OR “unwarranted” OR “unjustified” OR “over testing” OR “overtesting” OR “over-testing” OR “over diagnosis” OR “overdiagnosis” OR “over-diagnosis” OR “suboptimal” OR “sub-optimal” OR “poor quality” OR “poor-quality” OR “low utility” OR “limited utility” OR “poor utility” OR “not useful” OR “not helpful” OR “rarely useful” OR “rarely positive” OR “overtreatment” OR “over treatment” OR “over-treatment” | 807 580 |
| #2 | ae unit* OR "ae ward*" OR "aed*" OR "ae department" OR "a and e department*" OR "a and e service*" OR "a and e unit*" OR "a-e department*" OR "a-e unit*" OR "a-e ward" OR "accident and emergency service*" OR "accident and emergency ward*" OR "accident and emergency department*" OR "emergency ward*" OR "emergency room*" OR "casualty room*" OR "casualty department*" OR "casualty unit*" OR "emergicenter*" OR "emergicenter*" OR "emergency service*" OR "emergency room*" OR "emergency department*" OR "emergency hospital service*" OR "hospital emergency service*" OR "hospital emergency room*" OR "emergency outpatient unit*" OR "emergency medical service*" OR "emergency outpatient clinic*" OR "emergency care" OR "emergency medicine" OR "emergency visit*" OR "emergicenter*" OR "emergicenter*" OR "emergency health service*" OR "emergency medical service*" OR "medical emergency service*" OR "emergency unit*" OR "emergency clinician*" OR "emergency specialist*" OR "emergentologist*" OR "emergency room physician*" OR "emergency doctor*" OR "emergency medicine" OR "emergency care" OR "er physician*" OR "trauma patient*" OR "emergency patient*" OR "ed" OR "eds" OR "trauma centre*" OR "trauma center*" OR "trauma unit*" | 491 408 |
| #1 | "blood culture" OR "blood cultures" | 29 830 |

**Appendix 4.** Data charting tool used for scoping review

| Study ID | Author(s) | Year | Country | Study Design | Study Duration/ Monitoring Period | Setting | Sample Size | Number of blood cultures studied | Population characteristics | Aims/  Objectives | Criteria for LVBCs | Clinical features/  conditions associated with LVBCs |
| --- | --- | --- | --- | --- | --- | --- | --- | --- | --- | --- | --- | --- |
|  |  |  |  |  |  |  |  |  |  |  |  |  |

**Appendix 4.** Data charting tool used for scoping review (continued)

| Factors Influences/ Contributing to Ordering of LVBC | Estimated Prevalence/Frequency of LVBC Use | Positive Blood Culture Results | Clinically Significant Blood Culture Results (Impacting patient management) | Contamination rates/ False positive results | Harms/Consequences of LVBCs | Economic Implications | Key Findings | Limitations | Comments |
| --- | --- | --- | --- | --- | --- | --- | --- | --- | --- |
|  |  |  |  |  |  |  |  |  |  |
